# An Automated Blur Detection Method for Histological Whole Slide Imaging

**DOI:** 10.1371/journal.pone.0082710

**Published:** 2013-12-13

**Authors:** Xavier Moles Lopez, Etienne D'Andrea, Paul Barbot, Anne-Sophie Bridoux, Sandrine Rorive, Isabelle Salmon, Olivier Debeir, Christine Decaestecker

**Affiliations:** 1 Laboratories of Image, Signal processing and Acoustics (LISA), Université Libre de Bruxelles, Brussels, Belgium; 2 DIAPath - Center for Microscopy and Molecular Imaging, Université Libre de Bruxelles, Gosselies, Belgium; 3 Department of Pathology - Erasme Hospital, Université Libre de Bruxelles, Brussels, Belgium; University of Navarra, Spain

## Abstract

Whole slide scanners are novel devices that enable high-resolution imaging of an entire histological slide. Furthermore, the imaging is achieved in only a few minutes, which enables image rendering of large-scale studies involving multiple immunohistochemistry biomarkers. Although whole slide imaging has improved considerably, locally poor focusing causes blurred regions of the image. These artifacts may strongly affect the quality of subsequent analyses, making a slide review process mandatory. This tedious and time-consuming task requires the scanner operator to carefully assess the virtual slide and to manually select new focus points. We propose a statistical learning method that provides early image quality feedback and automatically identifies regions of the image that require additional focus points.

## Introduction

Whole slide scanners (WSSs) are novel devices that enable high-resolution imaging of an entire histological slide. The scanner used in the present study (Hamamatsu NanoZoomer) enables a resolution of 0.23 µm per pixel, corresponding to a 400X magnification. Furthermore, whole slide imaging (WSI) is achieved in only a few minutes, which enables image rendering of large-scale studies involving multiple immunohistochemistry (IHC) biomarkers [1]–[3]. While current scanners, such as the NanoZoomer, perform generally very well, whole slide images, usually denoted as virtual slides (VSs), can present blurred regions due to locally poor focusing. These artifacts should be avoided as much as possible because they can affect the quality of subsequent analysis, making a VS review process mandatory.

Most WSSs, such as the one used in this study, perform a focusing step at a set of locations (focusing points) prior to WSI. The number and position of these focusing points, which are either chosen by a human operator or automatically selected by the WSS, can strongly affect the resulting image quality because the 3D landscape of the slide is extrapolated from the chosen focusing points. While too few focusing points may result in a partially blurred VS, too many focusing points significantly increase the scanning time. We estimated that the scanner used in this study requires 2.8 s per focusing point. Therefore, a reasonable number of focusing points should be selected to ensure image quality for a majority of VSs. Typically, in our laboratory (DIAPath, CMMI), we used 9 focusing points for each 100-mm^2^ region. With this practical rule, approximately 75% of the slides are properly scanned (out of more than 2500 4- or 5-µm thick slides); these slides are obtained from various normal or pathological paraffin-embedded tissue samples stained with different standard techniques for brightfield microscopy, i.e., IHC and HE (hematoxylin-eosin), excluding fluorescence labeling, for which our scanner is not equipped. Under these conditions, the total scanning time is approximately 6 minutes for a large slide (of approximately 443 mm^2^) at 20X magnification. These data represent a good trade-off between scanning efficiency and time.

**Figure 1:**
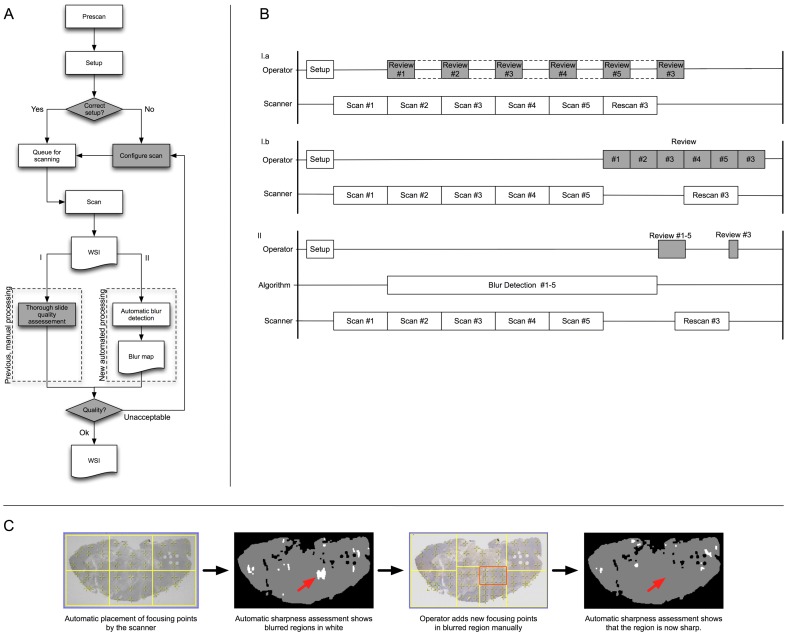
Complete scanning workflow and sequence of processes. A. Illustration of the complete scanning workflow. The first step is the "Prescan": the scanner starts by taking a low-resolution grayscale picture of all of the slides loaded by the operator. This grayscale image is used during the second step by the automatic "Setup" process: a predefined set of rules (known as the "profile") is applied to detect the tissue and to split large tissue regions in several regions with a maximum area of 100 mm^2^. Nine focusing points are then distributed across each piece of tissue to compute a median focusing plane for each small tissue piece. The automatic setup can fail if the tissue is very faint (tissue detection error) or if focusing points are placed on tissue folds or defects (focusing error). The operator is therefore asked to control the result of the profile and to correct it if necessary (by moving or adding focusing points). The slide is then ready to be scanned and is placed in the scanning queue (step 3). Steps 1 to 3 can occur concurrently, until all of the slides in the batch are queued. During step 4, the scanner scans the regions of the slides defined by the profile and produces the VS, which is saved on disk. The operator can then review the VS immediately while the scanner scans the next slide in the queue (see frame B I.a) or can let the scanner work until more VSs are ready for review (see frame B I.b). The manual review process (path I) consists of a thorough slide quality assessment. The method presented in this work (path II) consists of an automatic creation of a "blur map" showing the improperly focused regions. This method reduces the reviewing time of the operator and helps him (her) decide whether the VS is of acceptable quality. B. Sequence of operations for a hypothetical batch of five slides, with the time increasing from left to right along the horizontal axis. Without the assistance of an automated image quality assessment method (corresponding to path I in frame A), different review sequences are possible, ranging from continuous reviewing (I.a: the operator reviews each slide immediately after its acquisition) to batch reviewing (I.b: the operator waits until the entire batch is scanned before reviewing all of the slides). Continuous reviewing requires the operator to be continuously present during the scanning session (denoted by the dotted rectangle), while batch reviewing allows the operator to perform other tasks during imaging. The automated sharpness evaluation method presented in this work (corresponding to path II in frame A) results in the creation of blur maps during the scanning session, reducing the time needed for the manual review process (timeline II). In A-B, shaded boxes indicate the tasks carried out by the operator. C. Sequence of states of a slide on which the complete workflow (path II) is applied. The "Prescan" step defined six focusing planes, with nine focusing points each, and queued the slide for scanning. As soon as the VS is saved on disk, the automated sharpness evaluation method is applied and produces a "blur map", showing potential improperly scanned regions (red arrow on second image). The operator manually adds new focusing planes, and nine new focusing points are automatically distributed on each plane (the red rectangle targets the blurred region identified by the arrow on the blur map). The slide is scanned again, and when the new VS is available, the sharpness assessment method verifies the new file and shows that the blurred region disappeared (red arrow in the fourth image).

The complete scanning workflow process employed in our laboratory is presented in Figure 1 and is detailed in the figure caption. During the VS review process, the scanner operator has the following responsibilities: (i) assess the VS and (ii) either accept the slide if properly scanned or identify blurred regions for which new focus points must be added. This time-consuming task requires the scanner operator to carefully assess the entire slide image to identify poorly focused regions. Automated methods for VS quality assessment that identify blurred regions are therefore strongly needed.

Image quality assessment (IQA) and, more particularly, sharpness evaluation are well-studied fields of research [4]–[6]. Some of the proposed methods use a reference image to which the current image is compared [7]–[9]. However, this approach is unsuitable for VS quality assessment because such reference images are not available. Alternatively, most methods estimate image sharpness by characterizing the edges, contrast, or information content of the image [4], [10]. However, these methods relate to autofocusing; these approaches evaluate the sharpness for each z-position of the microscope stage during image acquisition and choose the position maximizing the sharpness measure as the correct focus plane [4], [5], [11]. In contrast, our application concerns sharpness assessment of a VS after (and not during) imaging (cf. Figure 1).

Studies related to VS sharpness assessment quantify sharpness in high-resolution subregions, i.e., tiles. This methodology identifies poorly focused subregions in the VS. The overall sharpness quality of the VS depends on the quality of each individual subregion. Zerbe *et al.* developed a distributed and scalable image analysis framework and evaluated the gain in processing time by applying it to a simple automatic quality assessment of VSs, i.e., by classifying each tile of an image into four sharpness categories: excellent quality, acceptable quality, to be reviewed and defective quality [12]. For this task, the authors used the well-known Tenengrad function to characterize the image sharpness [13]. Haralick features (e.g., contrast and entropy computed in a gray-level co-occurrence matrix) have also been used as VS quality factors [10]. Recently, Hashimoto *et al.* developed an image quality evaluation method based on a linear combination of image sharpness and noise measurements [15]. This method evaluated a quality index based on either an objective evaluation score, determined by comparing a perfect reference image to its artificially degraded version, or a subjective score given by human observers. These objective or subjective scores were then used as targets to train a regression model. The method efficiency was evaluated only on small regions sampled from a single hematoxylin-eosin (HE)-stained mouse embryo slide that was imaged by two different WSSs.

**Figure 2:**
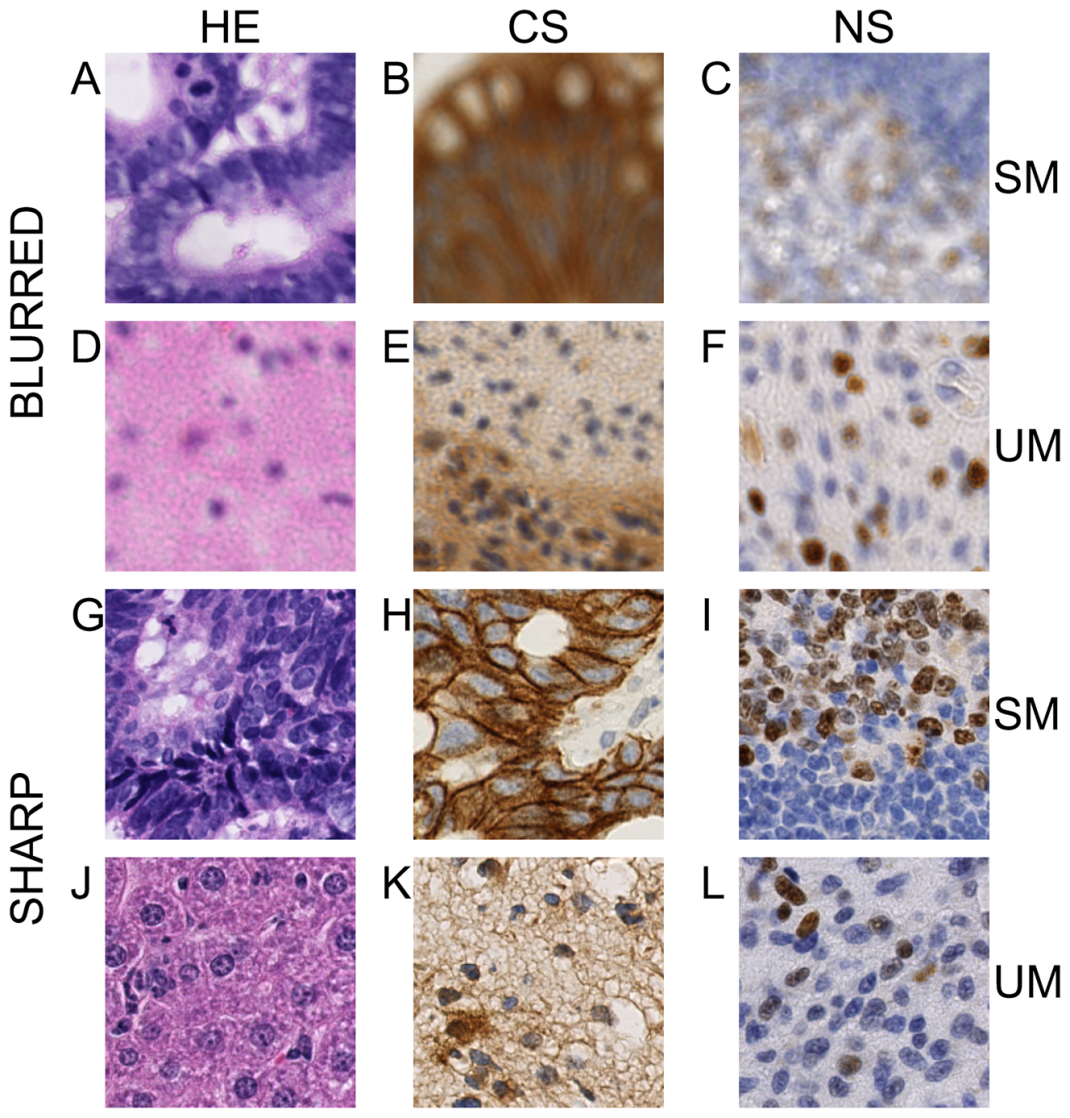
Examples of training samples. A - F. Examples of blurred samples. G - L. Examples of sharp samples. A, B, C and G, H, I show structured morphology (SM) samples. D, E, F and J, K, L show uniform morphology (UM) samples. Each column corresponds to a different staining pattern (HE, CS and NS, where CS includes membranous staining samples). These illustrations show typical tiles included in the training set.

Similar features were also used in [16] for cytological VS sharpness assessment. The authors proposed a complete automatic workflow that resolves the challenging problem of imaging single-layered cytological glass slides of sufficient quality. In-focus, single-layered imaging is achieved through an iterative process involving three steps: (1) semantic focusing, (2) imaging and automated quality assessment, and (3) saving the image or returning to (1) if the resulting VS is of poor quality. Semantic focusing (step 1) ensures that the focus points target cells and thus avoid the common artifacts encountered in a cytological glass slide. The quality assessment procedure (step 2) relies on cell segmentation and the computation of sharpness features on a sample of 200 isolated (or small clusters of) cells. Each cell or cluster of cells is then classified as sharp or blurred by a support vector machine (SVM). In step 3, the percentage of sharp cells is used to determine whether the slide should be rescanned. The general problem of correctly imaging a single-layered slide is more complex for cytological samples than for histological samples because of the higher variations in the 3D landscapes of cytological slides. However, estimating the local sharpness of a histological tile can involve particular problems due to the possible absence of tissue borders or histological structure edges in the evaluated region (see D - F, J - L in Figure 2). In contrast, the tiles from a cytological VS have a high probability of displaying borders of isolated cells or cell clusters in which the sharpness features are more sensitive. This is particularly true for the semantic focusing technique introduced in [16].

In addition to image sharpness or noise measurements, other IQA methods have been proposed such as visual codebook-based IQA and local dependency-based IQA [17]. These sophisticated methods aim to solve a general and complex problem known as non-distortion-specific IQA. This problem concerns images that can be altered by multiple distortions, such as JPEG compression artifacts, motion blur, focus blur, or the presence of white noise. However, focusing blur remains an important distortion that can affect VSs because other distortions are minimized by the technology used in modern WSSs. Therefore, the present study concentrates on the specific problem of sharpness evaluation for large images in which the sole distortion considered is a blur due to an incorrect (or suboptimal) focus during acquisition.

In the present study, we present various morphological and staining patterns that are regularly encountered in research activities applied to tissue-based biomarker evaluation [1], [2], [18], [19]. Figure 2 illustrates such a panel of tiles extracted from VSs, with some presenting blurred regions. To significantly reduce the operator time devoted to the VS review process, we developed a supervised classification approach using multiple sharpness features (which include or are similar to those used in [10], [12], [15], [16]) that identifies the blurred regions of a VS on a map. The map thus efficiently informs the scanner operator regarding where additional focus points are required (see Figure 1C).

In addition to tissue or slide defects (such as bubbles or tissue folds), local focusing during WSI can be altered by other characteristics. Indeed, local sharpness evaluation (conducted by the scanner in small regions centered on the focusing points) is more difficult for areas in which the tissue aspect is almost uniform and presents very little texture (e.g., in the absence of tissue borders, histological structure edges, or staining variations). We thus applied and evaluated our methodology on large datasets in order to cover different tissue aspects (in particular, the presence or absence of histological structures) and staining patterns (excluding fluorescence labeling) that can be encountered in HE and simple IHC VSs. IHC VSs can present nuclear, cytoplasmic and/or membranous expression of the targeted biomarkers. To the best of our knowledge, this study is the first to analyze such a panel of cases. This approach allows us to investigate whether the detection of blurred regions is impacted by the presence of edges caused by histological structures in addition to contrast modifications caused by IHC staining, which affect the high spatial frequencies used to measure image sharpness.

## Materials

### 1. Training samples

For training in the classification methods, 27 histological slides of 4 or 5 µm thickness were digitized at 20X magnification (0.46 µm/pixel) using the NanoZoomer HT Scan System (Hamamatsu Photonics, Japan). This scanner uses the line-scanning method (i.e., the slide is scanned by lines of 4096 pixels) and time delay integration (i.e., each line is scanned 64 times and averaged) to improve the signal to noise ratio. As mentioned in the introduction, the image sharpness is evaluated on high-resolution subregions of the VS, also denoted as tiles. We thus collected a total of 48,000 tiles of 200×200 pixels sampled from the 27 VSs taken at 20X magnification, which represented typical histological samples. The tile size enabled us to target specific VS regions, which presented different tissue and staining patterns and displayed either sharp or blurred areas.

As detailed in Table 1 and illustrated in Figure 2, tiles were sampled from VSs to account for two morphologic patterns: structured morphologies (SM, i.e., presenting well-defined histological structures such as colonic glands) and unstructured morphologies (UM, i.e., lacking clear histological structures such as brain tissue). These morphologic characteristics were combined with three staining patterns: HE, nuclear IHC (NS) and cytoplasmic IHC (CS), presenting variable expression levels of the targeted markers. We also included membranous staining patterns in the CS category (see Figure 2H), considering that (trans)membranous antigens, such as receptors, are also often expressed in the cytoplasm (due to internalization and protein trafficking).

**Table 1:** Tile origins.

	Tissue morphology	Hematoxylin and eosin (HE)	Nuclear IHC (NS)	Cytoplasmic IHC (CS)
			(tissue; IHC marker)	(tissue; IHC marker)
Blurred	SM	VS1: Endometrium	VS1: Salivary gland; Ki67	VS1: Colon; E-cadherin
		VS2: Parotid gland	VS2: Salivary gland; Ki67	VS2: Bladder; CD34
		VS3: Thyroid (Papillary carcinoma)	VS3: Lymph node; Ki67	
	UM	VS1: Brain	VS1: Brain; Ki67	VS1: Brain; Galectin-1
		VS2: Brain (Meningioma)	VS2: Brain; Ki67	VS2: Pancreas; Glucagon
			VS3*: Brain; Ki67	
Sharp	SM	VS1: Endometrium	VS1: Lymph node; Ki67	VS1: Colon; E-cadherin
		VS2: Colon (Adenocarcinoma)	VS2: Lymph node; Ki67	VS2: Colon; E-cadherin
	UM	VS1: Liver	VS1: Brain; p53	VS1: Brain; Galectin-1
		VS2: Joint (Synovitis)	VS2: Brain; p53	VS2: Brain; VEGFR-1
			VS3*: Brain; Ki67	

SM  =  structured morphology; UM  =  unstructured morphology; HE  =  hematoxylin-eosin; IHC  =  immunohistochemistry; NS  =  nuclear staining; CS  =  cytoplasmic and/or membranous staining. A set of 27 VSs was used with different tissue origins and different IHC biomarkers in the NS and CS categories. The VSs with an asterisk correspond to different tissue pieces of the same VS (one tissue piece was blurred while the other was sharp). The CS category includes biomarkers with membranous expression (e.g., CD34 and VEGFR-1). A total of 4,000 blurred and 4,000 sharp tiles were selected for each combination of morphology and staining pattern by targeting specific regions in the VSs (as illustrated in Figure 2).

The combination of morphologic and staining patterns resulted in six categories (see Table 1). For each category, we selected 4000 blurred and 4000 sharp tiles from a panel of VSs showing the patterns of interest (see Table 1). These different tile categories were included in the supervised dataset to best represent the large diversity of patterns that are encountered in HE and IHC slides.

### 2. Validation samples

To validate the method using independent slides, we selected another 97 VSs from our daily routine and imaged from 4- or 5-µm-thick tissue samples. For each slide, an expert randomly selected up to 20 blurred tiles and up to 20 sharp tiles. This new dataset of 3438 tiles (1462 from 39 HE slides and 1976 from 58 IHC slides) was then used as a validation set to estimate the performance of the classification algorithms (see section 3). This validation set included tiles that covered at least the six pattern categories included in the training data. The tiles were sampled from various tissue origins (e.g., skin, bladder, colon, pancreas, brain tumor, liver, and thyroid) and various IHC markers (e.g., KI67, glucagon, insulin, IGF1R, E-cadherin, CD79A, FHL2, GFAP, EGFR, HER2, etc.), including samples different from those encountered in the training set (see Table 1).

In addition to quantitative validation, we illustrate an application of this method to complete VSs (included in the independent set of 97 VSs), as detailed in section 3.3.

## Methods

### 1. Tile blur characterization

A set of 16 different blur features was determined per tile to locally evaluate the image sharpness of histological VSs.

In [15], the authors presented two image analysis metrics: sharpness (*SH*) and noise (*NO*). *SH* was computed for tiles that had been converted to grayscale (i.e., the Y component of the CIE XYZ color space). The gradient direction was determined for each pixel whose gradient was larger than a threshold value (determined by Otsu’s method [14]). The edge width (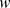) was defined as the distance (in pixels) between the local minimum and local maximum of the gradient. As described in [15], the edge width is modified as follows:
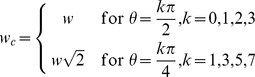



with *θ* being the edge direction sampled at 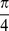. The edge width was then averaged across the tile to yield *SH*:
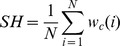



where 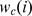 is the edge width of the *i*-th edge.

*NO* was computed by first subtracting a Gaussian-filtered (σ = 2) tile from the original tile. Using the resulting tile, *NO* was defined as the mean square of the minimum difference between each pixel and its eight nearest neighbors (in a 3×3 window). In contrast to *SH*, *NO* is the average of the *NO* value determined for the individual red, green and blue channels.
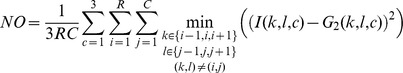



where *I(i, j, c)* and *G_2_(i, j, c)* are the intensity values of channel *c* at row *i* and column *j* of the original and Gaussian-filtered tile (with σ = 2), respectively. *R* and *C* are, respectively, the numbers of rows and columns in the tile (i.e., *R*  =  *C*  =  200 in our application).

We added three other features. The first two used the difference image, (*I* - *G_2_*), generated during *NO* computation and computed the mean value, or mean blur difference (*MBD*), and standard deviation, or standard blur difference (*SDBD*), across the difference image, respectively. The third feature is the mean value of the gradient, or the mean gradient magnitude (*MGM*), of a Gaussian-filtered tile (previously converted to gray-scale) with σ = 0.5 (*G_0.5_*). 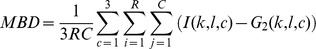


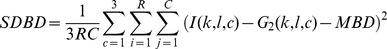


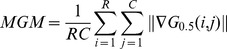



Similar to *SH* and *MGM*, the following features were computed for tiles converted to gray-scale. As suggested by [10], we computed a set of Haralick features, namely, the correlation and entropy features, computed on five gray-level co-occurrence matrices (GLCM). For a given tile *I* of size *n x m*, the GLCM computed for the offset (Δx, Δy) is defined as follows:
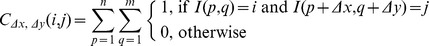



The normalized GLCM, labeled *P,* is obtained by dividing each element of *C* by the sum of all the elements of *C*:
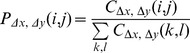



The contrast feature is then calculated as follows:
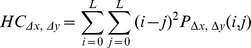



with *L* being the maximum possible gray value (for our images, *L*  =  255).

Similarly, the entropy is calculated as follows:
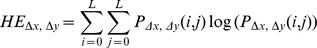



For each tile in our database, we computed the contrast and entropy values corresponding to the GLCMs for the set of offsets 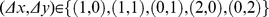, resulting in ten Haralick features.

For thoroughness, we also used the Tenengrad function (*TG_t_*), as described in [12]. *TG_t_* sums the square of the gradient values above a threshold *t* [13]. Because all of the tiles are the same size, the difference between *TG_0_* (i.e., *TG_t_* with *t*  =  0) and *MGM* is that the gradient is computed for the original and Gaussian-filtered tiles, respectively. To prevent a strong correlation between the *TG_t_* and *MGM* values, we altered the threshold of each tile by applying Otsu’s method to the squared norm of the gradient of the tile.

All of the features had larger values for sharp images compared to blurred images, with the exception of *SH*, which had larger values for blurred images [15]. All image features were computed in Python using SciPy [20] and the scikit-image image processing toolkit [21]. For performance purposes, we optimized the computation of the *SH, NO, MBD, SDBD, MGM, HE_Δx,Δy_* and *TG_t_* features by compiling the corresponding code to LLVM bytecode using the Numba library [22].

To compare the discriminatory abilities of the 16 features between sharp and blurred images, we used the **θ** measure, a non-parametric measure of effect size used to characterize the degree of separation of two distributions. This measure is easily estimated by *U/mn*, i.e., the Mann–Whitney *U* statistic divided by the product of the two sample sizes [23]. This normalized statistic ranges between 0 and 1, with values near 0.5 indicating similar distributions and values near 0 and 1 indicating strong separation. To simplify the comparison, we ranked the features by means of their discriminatory ability (or separation degree) evaluated by max(*U/mn*, 1-*U/mn*), which ranges between 0.5 (complete distribution overlap) and 1 (no overlap).

### 2. Tile classification and model selection

We used two different approaches of supervised classification: the decision tree (DT) approach, for which we tested three different split criteria, and the SVM approach which has been shown to be very efficient in cell classification [16] and for which we tested different kernels and hyper-parameter values. As detailed below, the DT approach enabled us to include feature selection in the classification process. Another advantage of this approach is that it generates relatively simple classification rules as a series of if-then statements, which are easy to implement in a decision process and are especially fast to apply in production (a particularly beneficial property). We also tested the SVM, which is a powerful, state-of-the-art algorithm with strong theoretical foundations that is particularly well adapted for binary classification in a numerical space [24]. This latter classifier enabled us to approximate the best results we could expect for our classification problems. As detailed below, for each classifier, we used a nested (5-fold x 5-fold) cross-validation to choose the hyper-parameters (inner 5-fold cross-validation) and to estimate the performance of the resulting model (outer 5-fold cross-validation), as is usually recommended to avoid overfitting [25].

The DT approach executes recursive partitioning of the feature space until it identifies subspaces in which the training cases predominantly belong to the same class (i.e., the majority class, which is allocated to the corresponding subspace). The DT was implemented using the classification tree module of the Statistica package (StatSoft, Tulsa, OK), except when the complete training set (i.e., grouping HE and IHC tiles) was considered. For this very large set (48,000 tiles), we used the QUEST Classification Tree software (version 1.10) [26]. These software packages allowed for the testing of different split criteria (for recursive partitioning), including univariate and multivariate split options. The univariate options are based either on an exhaustive search evaluated by the Gini index of node impurity [28] or on discriminant-based split criteria (based on ANOVA and *k*-means in the case of quantitative predictors, see [29]). The univariate split options resulted in a selection of the most discriminating features. The multivariate option determined a discriminant-based linear combination of all of the quantitative predictors provided for the model. In addition, minimal cost-complexity cross-validation pruning [28] was used to generate the “right-sized” tree for each split criterion. The complexity of a DT model is usually determined by its maximum depth, i.e., by the maximum number of splits required to determine the majority class in any region of the feature space. Finally, outer cross-validation (with inner cross-validation pruning performed on each training set) was used to evaluate the predictive performance of each algorithm.

The SVM approach aims to identify the boundary that represents the largest separation, or margin, between two classes. Initially developed for linear separation, the method was extended to solve non-linear problems by transforming the initial feature space (using kernels) into a new space in which the classes are (nearly) linearly separable [24]. Training a SVM classifier requires the selection of a kernel type (and its parameters) and a soft-margin parameter *C*, i.e., a penalty parameter for allowing some classification errors inside the margin (for nearly linearly separable classes in the transformed space). It can be shown that the SVM classification rule is a function of a subset of the training data that lie on the margin, i.e., the support vectors that determine the complexity of the class boundary implemented by the model [24]. The SVM was implemented using the SVC (Support Vector Classification) scikit-learn Python module [30]. A grid search was performed to identify the optimal hyper-parameters set by cross-validation. The grid search space was defined as

- kernels: { linear, rbf }

- *C*: {1, 2, 5, 10, 50, 100, 500, 1,000}

- 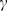: {10^−5^, 10^−4^, 10^−3^, 10^−2^, 10^−1^},

where 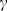 is the coefficient of the radial basis function (rbf) used as the kernel [24].

Outer cross-validation was used to evaluate the predictive performance of the optimal SVM classifier (defined by the set of hyper-parameter values that gave the best predictive performance in the inner cross-validation step).

As detailed in the results, the different classification algorithms were trained with different feature sets as inputs. We thus implemented a model selection process based on a trade-off between the predictive performance (evaluated by means of nested cross-validation, see above) and model complexity to favor both accurate and quick decision-making in the application phase. Candidate models were first selected on the basis of a tolerance of 0.5% applied to the best accuracy observed among all the generated models. Among these successful candidates, we kept the model(s) that required the lowest number of blur features for computation (which is the most time-consuming task during the application). If several models remained in the resulting selection, we then chose the model with the lowest complexity, evaluated by the maximum depth for the DT and by the number of support vectors for the SVM. This complexity criterion also prevented overfitting in the model selection [25].

### 3. Quality map production and VS annotation

**Figure 3:**
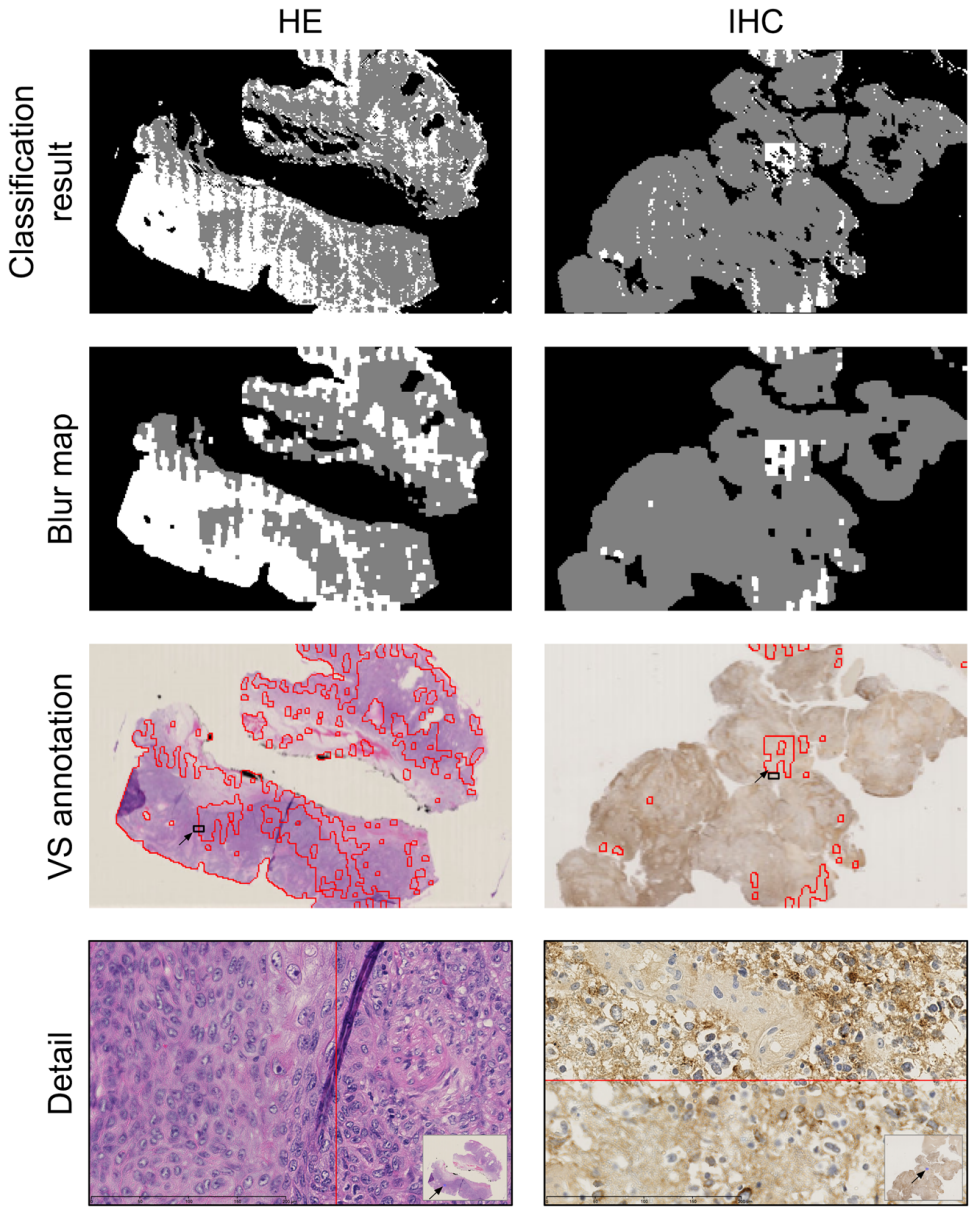
Application to complete HE and IHC VSs. The first row shows the raw classification results obtained by classifying all of the tiles constituting an HE and an IHC VS, by means of the HE-specialized DT and the IHC-specialized DT (see results for details), respectively. Gray or white pixels correspond to tiles classified as being sharp or blurred, respectively. The second row shows the final blur map obtained after grayscale morphological closing followed by an opening. In the third row, the borders of the blurred (gray) regions identified in the map are transferred as annotations on the VS view provided by our scanner viewer. Magnified details for the small rectangles (see arrows) are provided in the last line. In these two magnified fields (scale bar  =  200 µm), the red lines indicate the boundary between a sharp and a blurred region. The blurred region is located in the left-hand region for HE and at the bottom for IHC.

The map image was initialized by thresholding the image at 0.1X magnification of the entire slide. In the image, each pixel corresponds to a tile of 200×200 pixels at 20X magnification. To avoid computing the features and classifying tiles outside the tissue area, we first performed an Otsu thresholding procedure on the map image. Pixels corresponding to tiles outside versus within the tissue are were set to 0 versus 1. Tiles whose corresponding pixels were set to 1 were extracted at 20X magnification to compute the blur features and were then classified as being sharp or blurred. As illustrated in Figure 3, this classification resulted in a raw map showing black pixels (out-of-tissue area), grey pixels (sharp tiles) and white pixels (blurred tiles). This raw map may be difficult to interpret, especially in the case of scattered misclassification errors. Therefore, a post-processing tool, known as an alternate sequential filter [27] and consisting of a combination of a gray-scale morphological closing followed by a gray-scale morphological opening (with a 3×3 square as the structuring element), was applied to smooth the raw map and to ease the work of the operator. The contours of the blurred regions on the smoothed blur map were converted to an annotation compatible with NDP.View, the Hamamatsu annotation viewer. This conversion enables one to outline the blurred regions on the navigable VSs (Figure 3).

## Results

### 1. Feature analysis and selection

Table 2 shows the ranking of the features (in decreasing order) based on the discriminatory ability between blurred and sharp tiles. The discriminatory indices were evaluated by max(*U/mn*, (1–*U)/mn*) and ranged between 0.5 and 1 (see section 3.1). These indices and their rankings were computed for the complete training set as well as for the HE and IHC groups, taken separately. The results in Table 2 demonstrate high discriminatory ability for the *HC_Δx,Δy_* family, followed by the gradient-based features (*MGM* and *TG*), *NO* and the *HE_Δx,Δy_* family. *SDBD*, *SH* and *MBD* exhibit the weakest discriminatory ability. Interestingly, the feature ranking carried out for the complete training set is equivalent to the ranking average for the HE and IHC groups. This group distinction highlights some slight variations. For example, *TG* is more discriminatory in the HE tile group, whereas *MGM* is slightly more discriminatory in the IHC group.

**Table 2:** Discriminatory ability of the blur features.

Feature	Max(*U/mn*, 1-*U/mn*)			Rank			Mean Rank
	ALL	HE	IHC	ALL	HE	IHC	HE-IHC
*HC_11_*	0.99032	0.99441	0.99228	1	1	1	1
*HC_02_*	0.99027	0.99350	0.99134	2	3	2	2.5
*HC_20_*	0.98866	0.99423	0.99118	3	2	3	2.5
*HC_01_*	0.98777	0.99117	0.98821	4	4	5	4.5
*HC_10_*	0.98600	0.98941	0.99020	5	5	4	4.5
*MGM*	0.98186	0.98523	0.98386	6	7	6	6.5
*TG*	0.97710	0.98530	0.97831	7	6	8	7
*NO*	0.97123	0.97845	0.98025	8	8	7	7.5
*HE_10_*	0.94974	0.94504	0.95993	9	9	9	9
*HE_01_*	0.94926	0.94264	0.95882	10	10	10	10
*HE_11_*	0.94299	0.93918	0.95236	11	11	11	11
*HE_02_*	0.93177	0.92389	0.94154	12	14	12	13
*HE_20_*	0.93088	0.92497	0.94046	13	13	13	13
*SDBD*	0.81631	0.92933	0.88149	14	12	14	13
*SH*	0.78591	0.81661	0.77058	15	15	16	15.5
*MBD*	0.72515	0.74058	0.85791	16	16	15	15.5

*U*  =  Mann-Whitney statistic, *m*  =  *n*  =  blur and sharp sample sizes.

ALL  =  complete training set (*m*  =  *n*  =  24,000); HE  =  HE training set (*m*  =  *n*  =  8,000); IHC  =  IHC training set (*m*  =  *n*  =  16,000).

*HC*  =  Haralick contrast, *MGM*  =  mean gradient magnitude, *TG*  =  Tenengrad function, *NO*  =  noise, *HE*  =  Haralick entropy, *SDBD*  =  standard deviation of blur difference, *SH*  =  sharpness, *MBD*  =  mean blur difference.

**Figure 4:**
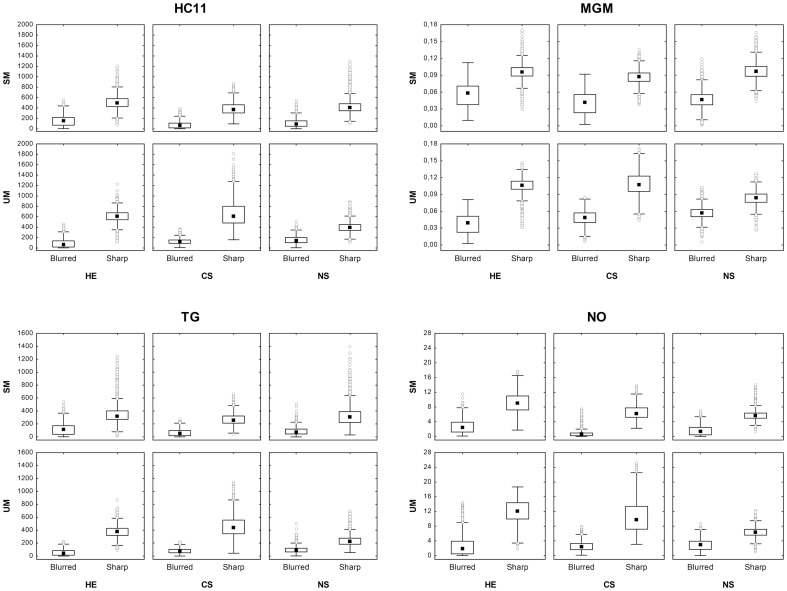
Distributions of *HC*_11_, *MGM*, *TG* and *NO* values. The feature values are computed for the training set. The results distinguish blurred and sharp tiles and are displayed by morphological patterns (structured vs. unstructured, i.e., SM vs. UM) in rows and staining types (HE, CS and NS) in columns. The data are shown by the following markers: median (black squares), 25–75% percentiles (boxes), non-outlier minimum and maximum (whiskers), and outliers (open dots).

Figure 4 details the data distributions of four discriminatory features across the six pattern categories constituting the training set. It should be noted that the level of discrimination between sharp and blurred images allowed by these features can vary from one category to another for a given staining group. These data demonstrate the benefit of including these different patterns in the dataset.

We also observed that the *HC_Δx,Δy_* features obtained for the different GLCMs were highly correlated among themselves (Spearman’s rank correlation coefficients *r_S_* > 0.95 for all training data). Even higher correlations were observed for the *HE_Δx,Δy_* features (*r_S_* > 0.98 for all training data). We thus selected the most discriminant feature in each family (i.e., *HC_11_* and *HE_10_*, cf. Table 2) for further analysis.

We ultimately kept a set of eight features (*HC_11_*, *MGM*, *TG*, *NO*, *HE_10_*, *SDBD*, *SH* and *MBD*) to train the classification models. Because some classification algorithms do not include a feature selection process (see section 3.2), we trained the models with different feature sets provided as input. To generate these feature sets, we implemented a backward elimination procedure, which is based on the discriminatory ability shown in Table 2 (i.e., beginning with the complete set and then removing the features, one by one, from the least to the most discriminatory feature).

### 2. Supervised classification models

While detailed results are provided in the supplementary data (Table S1), the top of Table 3 summarizes the characteristics of the DT classifiers selected on the basis of their performance (evaluated by nested cross-validation) and complexity (see section 3.2). This table describes the results obtained with and without consideration of the two staining groups (i.e., HE and IHC vs. ALL), together with the results pooled for the two staining groups (see row “Pooled”).

**Table 3:** Characteristics of the DT models selected on the basis of both their performance with the training set and their complexity and results obtained on the validation set.

Set	N tiles	DT algorithm	B -> S	S -> B	Accuracy	Sensitivity (blurred)	Features in DT model
Training							
HE	16000	Gini criterion	115	188	0.9811	0.9856	*HC_11_, MGM, TG, NO, HE_10_*
IHC	32000	Gini criterion	567	592	0.9638	0.9646	*HC_11_, MGM, TG*
Pooled (HE + IHC)	48000		682	780	0.9695	0.9716	*(HC_11_, MGM, TG, NO, HE_10_)*
ALL	48000	Gini criterion	927	1006	0.9597	0.9614	*HC_11_, MGM, TG, NO*
Validation							
HE	1462	HE DT	27	126	0.8953	0.9632	
IHC	1976	IHC DT	99	104	0.8973	0.8919	
Pooled (HE + IHC)	3438	HE DT or IHC DT	126	230	0.8965	0.9236	
ALL	3438	ALL DT	211	173	0.8883	0.8720	

Classification performances are shown in terms of accuracy (the percentage of correctly classified tiles) and sensitivity (the percentage of blurred tiles correctly classified as blurred by the DT). Columns "B -> S" and "S -> B" show the number of false negative decisions (i.e., the blurred tiles classified as being sharp) and the number of false positive decisions (i.e., the sharp tiles classified as being blurred), respectively. Concerning DT training, the classification performances resulted from a nested cross-validation (5-fold x 5-fold) carried out either on the HE and IHC groups separately or on all of the training data (see column “Set”). Columns "DT algorithm" and "Features in DT model" describe the choices resulting from the model selection method (i.e., DT split criterion and selected features, respectively). All of the results leading to the presented selections are detailed in the supplementary data (Table S1). Concerning the quantitative validation step, the models selected during the training step (see column “DT algorithm”) were applied to independent sets of tiles (see column “Set”). The resulting classification performances are indicated as for training. The number of tiles in each set is specified in column “N tiles”. Rows “Pooled” indicate the results obtained by pooling the two previous rows (HE and IHC) to allow for comparisons with the results in rows “ALL” below.

The accuracies (i.e., the percentage of correctly classified tiles) of the HE and IHC classifiers were 98.11% and 96.38%, respectively. Training only one classifier on all the data resulted in a global drop of approximately 1% in accuracy and sensitivity (i.e., the percentage of blurred tiles correctly classified as blurred by the classifier) (see lines “ALL” and “HE + IHC Pooled” in Table 3). All of these classifiers (selected for providing a good trade-off between accuracy and complexity) were trained with the Gini (univariate) split criterion. The difference between the HE and IHC classifier accuracies can be explained by the higher heterogeneity encountered in IHC slides. In addition, HE staining helps to introduce contrast in VSs because hematoxylin colors all cell nuclei in blue, whereas eosin stains most of the cell cytoplasm (and other extracellular eosinophilic structures) in various shades of pink (or even red). In contrast, IHC VSs mix IHC staining (with variable locations) with hematoxylin counterstaining, making the cell cytoplasm nearly invisible in areas in which the IHC biomarker is not expressed (see Figure 2). This difference may explain the increased difficulty for distinguishing between blurred and sharp tiles in the IHC group.

We also tested the impact of training more specialized classifiers for IHC VSs, one for nuclear staining and another for cytoplasmic and/or membranous staining (denoted NS and CS categories in Table 1, respectively). The accuracies of the selected classifiers (see Table S1) were 96.68% (NS) and 97.34% (CS). Their pooled performance (97.01%) was slightly higher than that of the IHC classifier (see Table 3). However, the specialized NS classifier required a computation of the eight blur features for the tiles (see Table S1), instead of only three for the generic IHC classifier (see Table 3). In addition, this generic classifier requires no distinction between staining locations. This latter property is interesting for future applications because subcellular antigen locations may vary in a single VS (e.g., due to possible protein translocation between the cell cytoplasm and the cell nucleus, as we observed in a previous study [31]).

The six pattern categories constituting the training set enabled us to identify which pattern(s) contributed the most to the classification errors. When using the HE-specialized DT to classify the HE tile group, the SM (structured morphology) pattern exhibited the largest (but acceptable) misclassification rate (slightly more than 2%, with a similar impact on the sensitivity), whereas the tiles presenting an UM (unstructured morphology) pattern were nearly always correctly classified (less than 1% of error) with a sensitivity near 100% for the blurred tiles. It should also be noted that the unspecialized DT strongly deteriorated the results for the SM-HE tiles (classification accuracy of approximately 93% and sensitivity of approximately 88%), whereas the results for the UM-HE tiles remained nearly similar (with a slight decrease in specificity). In the IHC group, the IHC-specialized classifier had the greatest difficulty in correctly classifying tiles presenting a combination of the UM pattern and the nuclear staining (NS) location (approximately 6% of errors relatively equidistributed between sharp and blurred tiles). In contrast, the UM-CS combination presented the best classification rate (near 99%). The two other categories (SM-NS and SM-CS) presented intermediate error contributions. In contrast to the HE group, the unspecialized classifier provided similar performances for all categories in the IHC group, without particular deterioration.

For completeness, we compared the DT approach to the SVM, a state-of-the-art classification approach. The SVM showed slightly improved performances compared to the DT approach, at the cost of a higher model complexity. Indeed, the best SVM classifiers, which were separately trained on the HE and IHC groups (with all of the features as input), exhibited classification accuracies of 99.06% and 97.23%, respectively. However, these two SVM models (kernel: rbf, C = 1000 and 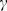 = 0.0001) required the computation of dot products with 510 and 2216 support vectors, respectively, to classify a tile. Comparatively, the most accurate DT models, which were generated with all of the features as input, provided similar performances (i.e., 98.56% and 96.63% classification accuracy, respectively) but with maximal DT depths of only 6 and 8, respectively (see Table S1). These results imply that DT algorithms provide very simple classification rules, which allow for accurate and quick decision-making in tile classification.

In view of all these results, we therefore chose to use the DT approach for blur detection application and selected the two generic DT classifiers, one for each staining type (HE vs. IHC), as described in Table 3. The resulting models were tested on an independent set of VSs, as described in section 4.3.

### 3. Validation on an independent tile set and application to entire VSs

We first verified that the ability to discriminate blur features was conserved in the validation set of 3438 tiles. We obtained a ranking similar to that previously observed for the training set (see Table 2), with a decrease in the discriminatory indices, i.e., between 0.96109 for HC_11_ and 0.65573 for *MBD* (evaluated on a much smaller dataset than the training set).

Table 3 (at the bottom) details the results obtained with the selected HE and IHC DTs on this completely independent dataset extracted from our daily routine in WSI and including samples vey different from those used for classifier training (see section 2.2). These results show an accuracy of near 90% for the two DTs (with a better sensitivity in the case of the HE DT). As previously observed on the training set, these performances decreased when the unspecialized (ALL) DT was used (in particular, the sensitivity for the HE tiles decreased to 82.54%).

In the final application (see Figure 3), all of the tiles constituting a VS should be classified. A post-processing step that considers the spatial consistency of the resulting tile labels (i.e., sharp or blurred) is then applied, withdrawing scattered classification errors. We found that this complete process takes approximately 15 minutes per image (averaged for the 97 slides of the validation dataset). After transferring blurry region borders as annotations in the VS view, the operator then only needs to verify consistently blurry regions to identify those that require additional focusing points to improve the VS quality (as shown in Figure 1C). This information may thus strongly reduce the manual reviewing time by focusing the operator’s attention on regions identified as blurry and for which the addition of new focusing points could be beneficial for locally improving the VS sharpness. If the operator confirms that the regions outlined in the VS are blurry, he/she can redistribute the focusing planes and the focusing points around the blurry regions and can then queue the slide for a rescan (see Figure 1C).

## Discussion and Conclusions

Compared to the previous studies mentioned in the introduction [10], [12], [15], [16], the present study achieved the following objectives:

- considered various morphological and staining patterns usually encountered in histological samples,
- developed an extended set of descriptive features and evaluated their effective contribution, and
- evaluated the systematic performance of various classification algorithms (i.e., DT and SVM approaches, various split criteria for DT and kernels for SVM) using different feature sets as inputs.

A statistical analysis of the blur features showed that the most discriminatory features were the Haralick contrasts, followed by the gradient-based features (*MGM*, *TG*), *NO*, and, finally, the Haralick entropies. These properties were observed when considering the entire dataset and persisted in each of the IHC and HE groups (in the training and validation sets). This feature ranking resulted from univariate analyses and was confirmed from a multivariate point of view by our classification results. Indeed, the less discriminatory features (such as *SBD*, *SH* and *MBD*) were systematically omitted in the best DT models. In addition, *NO* was also discarded for classifying IHC VSs. Consequently, a method that uses only the *NO* and *SH* features (such as in [15]) should be less effective in distinguishing blurred from sharp image regions, in particular in IHC VSs.

In the present study, we opted for a classification approach in contrast to Hashimoto et al. who developed a regression model [15]. This latter model requires training data with a quantitative score to characterize tile sharpness. A thresholding step should thus be added to distinguish between blurred and sharp tiles. We preferred a more direct approach based on supervised tile classification ("sharp" vs. "blurred"). Our approach directly provides a binary result for each tile, which is easier and more rapid to interpret by the operator than the score provided by a regression model. As discussed below, our method includes an additional post-processing step that considers the spatial consistency of the resulting tile labels.

Concerning the classification algorithms, we observed no significant performance improvement when using the SVM, a powerful and state-of-the-art algorithm, when compared to the DT approach. For this reason, we deemed it unnecessary to test other state-of-the-art classification algorithms, such as the weak classifier assembly [32] or random forest [33]. Indeed, these latter algorithms generate more complex classification rules than DT, whereas the expected performance improvement appeared to be low. An increase in rule complexity would require more computational resources and would slow the application phase (which requires the classification of thousands of tiles).

Our analysis identified two efficient DT classifiers, one for HE tiles and one for IHC tiles, based on a reduced number of features (four for HE and three for IHC). We also observed that some tissue patterns induced more difficulties in blurred tile detection. Hence, the IHC tiles were more difficult to classify than the HE tiles, with a particular contribution of UM-NS patterns (i.e., nuclear IHC staining in unstructured tissue regions) to classification errors. These data illustrate the effects of the combination of tissue morphology and staining patterns on the ability to detect blurred image regions. The DT results also indicate that different types of blur features (such as those related to the co-occurrence matrix and the image gradient) are required to accurately detect blurred regions in VSs.

The application to complete VSs consists of generating a raw classification map by using the appropriate (HE or IHC) DT. This raw map is then submitted to post-processing to generate a smoothed blur map that considers spatial consistency. Blurred regions are then outlined on the (navigable) VSs, strongly easing the VS review process. We designed the blur detection method to begin with a batch scanning process. Blur detection of one slide can be performed during the scanning of other slides. The WSS operator does not have to wait until the end of the scan (of dozens of slides) before reviewing each slide and rescanning if necessary. To further automate the workflow, we also plan to integrate our method with the scanner control routines through the use of the application programming interface (API), such as that described in [16]. We could enhance the first focusing step by implementing semantic focusing that can detect tissue folds [34]. Finally, we concentrated the present study on HE and simple IHC VSs. Extensions to special histological staining and double IHC are also included in future research plans.

### Code Availability

The source code for the Python tool presented in this work can be found at:

https://bitbucket.org/diapath/sharpa-lite.

## Supporting Information

Table S1 **Characteristics of the DT models produced during the training phase.** For each training set of tiles we ranked the DT models according to their respective accuracy. Column "Set" gives the name of the dataset used (HE  =  hematoxylin and eosin, NS  =  nuclear staining, CS  =  cytoplasmic staining, IHC  =  immunohistochemistry, ALL  =  complete training set) and column "N" the number of tiles in the dataset. Column “DT-Algorithm” indicates the split criterion used to create the DT (Discri multi  =  discriminant-based multivariate split, Discri uni  =  discriminant-based univariate split, Gini  =  exhaustive search for univariate split evaluated by the Gini index of node impurity). Column “Features” lists the set of features used for training (*HC*  =  Haralick contrast, *MGM*  =  mean gradient magnitude, *TG*  =  Tenengrad function, *NO*  =  noise, *HE*  =  Haralick entropy, *SDBD*  =  standard deviation of blur difference, *SH*  =  sharpness, *MBD*  =  mean blur difference). Columns "B -> S" and "S -> B" show the number of false negative decisions (i.e., the blurred tiles classified as being sharp) and the number of false positive decisions (i.e., the sharp tiles classified as being blurred), respectively. The classification performances provided in column “Accuracy” resulted from a nested cross-validation (5-fold x 5-fold) on each training set. For each dataset, we examined the DT models whose accuracy was higher than the maximum accuracy minus 0.005. DTs satisfying that condition are surrounded by a bounding box. For these models, additional columns detail the number of nodes in the tree (N nodes in tree), the number of tests in the tree (N tests in tree), the maximum depth of the tree (Tree depth), the number of features selected in the final DT model and the corresponding set of ignored features. The DT model finally selected is outlined in green (see main text for details).
(PDF)
