# Supplementary material for: An Automated Blur Detection Method for Histological Whole Slide Imaging
Source: PLoS One. 2013 Dec 13;8(12):e82710. doi: 10.1371/journal.pone.0082710 (PMC3862630; doi:10.1371/journal.pone.0082710)
Supplement: Table S1 — Characteristics of the DT models produced during the training phase. For each training set of tiles we ranked the DT models according to their respective accuracy. Column "Set" gives the name of the dataset used (HE = hematoxylin and eosin, NS = nuclear staining, CS = cytoplasmic staining, IHC = immunohistochemistry, ALL = complete training set) and column "N" the number of tiles in the dataset. Column “DT-Algorithm” indicates the split criterion used to create the DT (Discri multi = discriminant-based multivariate split, Discri uni = discriminant-based univariate split, Gini = exhaustive search for univariate split evaluated by the Gini index of node impurity). Column “Features” lists the set of features used for training (HC = Haralick contrast, MGM = mean gradient magnitude, TG = Tenengrad function, NO = noise, HE = Haralick entropy, SDBD = standard deviation of blur difference, SH = sharpness, MBD = mean blur difference). Columns "B -> S" and "S -> B" show the number of false negative decisions (i.e., the blurred tiles classified as being sharp) and the number of false positive decisions (i.e., the sharp tiles classified as being blurred), respectively. The classification performances provided in column “Accuracy” resulted from a nested cross-validation (5-fold x 5-fold) on each training set. For each dataset, we examined the DT models whose accuracy was higher than the maximum accuracy minus 0.005. DTs satisfying that condition are surrounded by a bounding box. For these models, additional columns detail the number of nodes in the tree (N nodes in tree), the number of tests in the tree (N tests in tree), the maximum depth of the tree (Tree depth), the number of features selected in the final DT model and the corresponding set of ignored features. The DT model finally selected is outlined in green (see main text for details). (PDF) [file pone.0082710.s001.pdf]

Set	DT-Algorithm	N	N features in training	Features	B -> S	S -> B	Accuracy	Sensitivity	Rank	N nodes in tree	N tests in tree	Tree depth	N features in tree	Ignored features
HE	Discri multi	16000	8	HC11, MGM, TG, NO, HE10, SDBD, SH, MBD	116	115	0.9856	0.9855	1	27	13	6	8	
HE	Discri uni	16000	8	HC11, MGM, TG, NO, HE10, SDBD, SH, MBD	94	164	0.9839	0.9883	2	39	19	8	7	SH,
HE	Gini	16000	8	HC11, MGM, TG, NO, HE10, SDBD, SH, MBD	104	158	0.9836	0.9870	3	37	18	8	7	SDBD,
HE	Discri multi	16000	6	HC11, MGM, TG, NO, HE10, SDBD	166	102	0.9833	0.9793	4	39	19	15	6	
HE	Discri multi	16000	7	HC11, MGM, TG, NO, HE10, SDBD, SH	170	105	0.9828	0.9788	5	35	17	13	7	
HE	Discri uni	16000	7	HC11, MGM, TG, NO, HE10, SDBD, SH	104	174	0.9826	0.9870	6	39	19	8	6	SH
HE	Discri uni	16000	6	HC11, MGM, TG, NO, HE10, SDBD	104	174	0.9826	0.9870	6	39	19	8	6	
HE	Gini	16000	6	HC11, MGM, TG, NO, HE10, SDBD	114	171	0.9822	0.9858	8	35	17	6	6	
HE	Gini	16000	7	HC11, MGM, TG, NO, HE10, SDBD, SH	117	169	0.9821	0.9854	9	37	18	7	7	
HE	Gini	16000	5	HC11, MGM, TG, NO, HE10	115	188	0.9811	0.9856	10	23	11	6	5	
HE	Discri multi	16000	5	HC11, MGM, TG, NO, HE10	141	177	0.9801	0.9824	11					
HE	Discri uni	16000	5	HC11, MGM, TG, NO, HE10	123	214	0.9789	0.9846	12					
HE	Discri multi	16000	4	HC11, MGM, TG, NO	175	171	0.9784	0.9781	13					
HE	Gini	16000	4	HC11, MGM, TG, NO	154	197	0.9781	0.9808	14					
HE	Discri uni	16000	4	HC11, MGM, TG, NO	119	233	0.9780	0.9851	15					
HE	Discri multi	16000	3	HC11, MGM, TG	197	163	0.9775	0.9754	16					
HE	Discri uni	16000	3	HC11, MGM, TG	154	227	0.9762	0.9808	17					
HE	Gini	16000	3	HC11, MGM, TG	167	254	0.9737	0.9791	18					
HE	Discri multi	16000	2	HC11, MGM	213	236	0.9719	0.9734	19					
HE	Discri uni	16000	2	HC11, MGM	209	256	0.9709	0.9739	20					
HE	Gini	16000	2	HC11, MGM	186	287	0.9704	0.9768	21					
HE	Discri uni	16000	1	HC11	319	193	0.9680	0.9601	22					
HE	Discri multi	16000	1	HC11	319	193	0.9680	0.9601	22					
HE	Gini	16000	1	HC11	290	224	0.9679	0.9638	24					
HE	Discri uni	16000	1	MGM	288	483	0.9518	0.9640	25					
HE	Discri multi	16000	1	MGM	288	483	0.9518	0.9640	25					
HE	Gini	16000	1	MGM	267	511	0.9514	0.9666	27					
NS	Discri multi	16000	8	HC11, MGM, TG, NO, HE10, SDBD, SH, MBD	285	246	0.9668	0.9644	1	21	10	7	8	
NS	Discri multi	16000	6	HC11, MGM, TG, NO, HE10, SDBD	324	292	0.9615	0.9595	2					
NS	Discri multi	16000	7	HC11, MGM, TG, NO, HE10, SDBD, SH	350	268	0.9614	0.9563	3					
NS	Discri multi	16000	5	HC11, MGM, TG, NO, HE10	333	292	0.9609	0.9584	4					
NS	Gini	16000	8	HC11, MGM, TG, NO, HE10, SDBD, SH, MBD	321	330	0.9593	0.9599	5					
NS	Gini	16000	7	HC11, MGM, TG, NO, HE10, SDBD, SH	315	348	0.9586	0.9606	6					
NS	Discri uni	16000	8	HC11, MGM, TG, NO, HE10, SDBD, SH, MBD	390	282	0.9580	0.9513	7					
NS	Discri multi	16000	4	HC11, MGM, TG, NO	359	318	0.9577	0.9551	8					
NS	Discri uni	16000	7	HC11, MGM, TG, NO, HE10, SDBD, SH	414	270	0.9573	0.9483	9					
NS	Discri multi	16000	3	HC11, MGM, TG	397	290	0.9571	0.9504	10					
NS	Discri uni	16000	4	HC11, MGM, TG, NO	382	308	0.9569	0.9523	11					
NS	Discri uni	16000	3	HC11, MGM, TG	376	317	0.9567	0.9530	12					
NS	Discri uni	16000	2	HC11, MGM	376	317	0.9567	0.9530	12					
NS	Discri multi	16000	2	HC11, MGM	393	316	0.9557	0.9509	14					
NS	Gini	16000	6	HC11, MGM, TG, NO, HE10, SDBD	318	399	0.9552	0.9603	15					
NS	Discri uni	16000	6	HC11, MGM, TG, NO, HE10, SDBD	441	280	0.9549	0.9449	16					
NS	Discri uni	16000	5	HC11, MGM, TG, NO, HE10	427	297	0.9548	0.9466	17					
NS	Gini	16000	4	HC11, MGM, TG, NO	346	385	0.9543	0.9568	18					
NS	Gini	16000	5	HC11, MGM, TG, NO, HE10	347	385	0.9543	0.9566	19					
NS	Gini	16000	3	HC11, MGM, TG	354	384	0.9539	0.9558	20					
NS	Gini	16000	2	HC11, MGM	350	391	0.9537	0.9563	21					
NS	Discri uni	16000	1	HC11	465	286	0.9531	0.9419	22					
NS	Discri multi	16000	1	HC11	465	286	0.9531	0.9419	22					
NS	Gini	16000	1	HC11	394	361	0.9528	0.9508	24					
NS	Discri uni	16000	1	MGM	528	621	0.9282	0.9340	25					
NS	Discri multi	16000	1	MGM	528	621	0.9282	0.9340	25					
NS	Gini	16000	1	MGM	521	648	0.9269	0.9349	27					
CS	Discri multi	16000	8	HC11, MGM, TG, NO, HE10, SDBD, SH, MBD	149	216	0.9772	0.9814	1	21	10	7	8	
CS	Gini	16000	8	HC11, MGM, TG, NO, HE10, SDBD, SH, MBD	149	230	0.9763	0.9814	2	25	12	6	6	SDBD, MBD
CS	Gini	16000	7	HC11, MGM, TG, NO, HE10, SDBD, SH	180	207	0.9758	0.9775	3	25	12	6	6	SDBD
CS	Discri uni	16000	6	HC11, MGM, TG, NO, HE10, SDBD	231	176	0.9746	0.9711	4	33	16	7	6	
CS	Discri uni	16000	8	HC11, MGM, TG, NO, HE10, SDBD, SH, MBD	221	187	0.9745	0.9724	5	33	16	6	7	SH
CS	Discri uni	16000	7	HC11, MGM, TG, NO, HE10, SDBD, SH	221	187	0.9745	0.9724	5	33	16	7	6	SH
CS	Gini	16000	6	HC11, MGM, TG, NO, HE10, SDBD	176	235	0.9743	0.9780	7	25	12	6	6	
CS	Discri multi	16000	7	HC11, MGM, TG, NO, HE10, SDBD, SH	197	221	0.9739	0.9754	8	17	8	5	7	
CS	Discri multi	16000	6	HC11, MGM, TG, NO, HE10, SDBD	201	220	0.9737	0.9749	9	17	8	5	6	
CS	Gini	16000	5	HC11, MGM, TG, NO, HE10	178	245	0.9736	0.9778	10	21	10	5	5	
CS	Gini	16000	4	HC11, MGM, TG, NO	177	248	0.9734	0.9779	11	15	7	4	4	
CS	Discri uni	16000	5	HC11, MGM, TG, NO, HE10	206	261	0.9708	0.9743	12					
CS	Discri uni	16000	4	HC11, MGM, TG, NO	198	274	0.9705	0.9753	13					
CS	Discri multi	16000	5	HC11, MGM, TG, NO, HE10	220	256	0.9703	0.9725	14					
CS	Discri multi	16000	3	HC11, MGM, TG	216	284	0.9688	0.9730	15					
CS	Gini	16000	3	HC11, MGM, TG	279	225	0.9685	0.9651	16					
CS	Discri multi	16000	4	HC11, MGM, TG, NO	268	243	0.9681	0.9665	17					
CS	Discri multi	16000	2	HC11, MGM	174	346	0.9675	0.9783	18					
CS	Discri uni	16000	3	HC11, MGM, TG	253	277	0.9669	0.9684	19					
CS	Gini	16000	2	HC11, MGM	236	311	0.9658	0.9705	20					
CS	Discri uni	16000	2	HC11, MGM	286	273	0.9651	0.9643	21					
CS	Gini	16000	1	HC11	208	361	0.9644	0.9740	22					
CS	Discri uni	16000	1	HC11	315	275	0.9631	0.9606	23					
CS	Discri multi	16000	1	HC11	315	275	0.9631	0.9606	23					
CS	Discri uni	16000	1	MGM	382	442	0.9485	0.9523	25					
CS	Discri multi	16000	1	MGM	382	442	0.9485	0.9523	25					
CS	Gini	16000	1	MGM	347	482	0.9482	0.9566	27					
IHC	Gini	32000	7	HC11, MGM, TG, NO, HE10, SDBD, SH	511	564	0.9664	0.9681	1	39	19	8	6	NO
IHC	Gini	32000	8	HC11, MGM, TG, NO, HE10, SDBD, SH, MBD	557	523	0.9663	0.9652	2	39	19	8	7	NO
IHC	Gini	32000	6	HC11, MGM, TG, NO, HE10, SDBD	529	560	0.9660	0.9669	3	43	21	9	6	

IHC	Discri multi	32000	8	HC11, MGM, TG, NO, HE10, SDBD, SH, MBD	559	539	0.9657	0.9651	4	31	15	5	8	
IHC	Gini	32000	4	HC11, MGM, TG, NO	549	550	0.9657	0.9657	5	23	11	8	4	
IHC	Discri multi	32000	7	HC11, MGM, TG, NO, HE10, SDBD, SH	553	559	0.9653	0.9654	6	31	15	7	7	
IHC	Discri multi	32000	6	HC11, MGM, TG, NO, HE10, SDBD	587	525	0.9653	0.9633	6	25	12	5	6	
IHC	Gini	32000	5	HC11, MGM, TG, NO, HE10	572	556	0.9648	0.9643	8	19	9	6	5	
IHC	Gini	32000	3	HC11, MGM, TG	567	592	0.9638	0.9646	9	17	8	6	3	
IHC	Discri multi	32000	5	HC11, MGM, TG, NO, HE10	569	619	0.9629	0.9644	10	11	5	3	5	
IHC	Discri multi	32000	4	HC11, MGM, TG, NO	538	654	0.9628	0.9664	11	17	8	4	4	
IHC	Discri multi	32000	3	HC11, MGM, TG	505	698	0.9624	0.9684	12	13	6	4	3	
IHC	Discri uni	32000	3	HC11, MGM, TG	685	576	0.9606	0.9572	13					
IHC	Discri multi	32000	2	HC11, MGM	575	693	0.9604	0.9641	14					
IHC	Discri uni	32000	2	HC11, MGM	639	661	0.9594	0.9601	15					
IHC	Discri uni	32000	8	HC11, MGM, TG, NO, HE10, SDBD, SH, MBD	672	634	0.9592	0.9580	16					
IHC	Discri uni	32000	7	HC11, MGM, TG, NO, HE10, SDBD, SH	672	634	0.9592	0.9580	16					
IHC	Discri uni	32000	5	HC11, MGM, TG, NO, HE10	665	647	0.9590	0.9584	18					
IHC	Gini	32000	2	HC11, MGM	684	628	0.9590	0.9573	18					
IHC	Discri uni	32000	6	HC11, MGM, TG, NO, HE10, SDBD	709	615	0.9586	0.9557	20					
IHC	Discri uni	32000	4	HC11, MGM, TG, NO	709	615	0.9586	0.9557	20					
IHC	Discri uni	32000	1	HC11	771	574	0.9580	0.9518	22					
IHC	Discri multi	32000	1	HC11	771	574	0.9580	0.9518	22					
IHC	Gini	32000	1	HC11	641	717	0.9576	0.9599	24					
IHC	Discri uni	32000	1	MGM	901	1064	0.9386	0.9437	25					
IHC	Discri multi	32000	1	MGM	901	1064	0.9386	0.9437	25					
IHC	Gini	32000	1	MGM	902	1077	0.9382	0.9436	27					

ALL	Gini	48000	8	HC11, MGM, TG, NO, HE10, SDBD, SH, MBD	942	781	0.9641	0.9608	1	45	22	8	7	HE10
ALL	Discri multi	48000	8	HC11, MGM, TG, NO, HE10, SDBD, SH, MBD	986	757	0.9637	0.9589	2	57	28	13	8	
ALL	Discri multi	48000	7	HC11, MGM, TG, NO, HE10, SDBD, SH	1094	694	0.9628	0.9544	3	51	25	9	7	
ALL	Gini	48000	7	HC11, MGM, TG, NO, HE10, SDBD, SH	1000	797	0.9626	0.9583	4	51	25	8	7	
ALL	Discri uni	48000	8	HC11, MGM, TG, NO, HE10, SDBD, SH, MBD	830	1015	0.9616	0.9654	5	43	21	9	7	HE10
ALL	Gini	48000	6	HC11, MGM, TG, NO, HE10, SDBD	1026	824	0.9615	0.9573	6	47	23	8	6	
ALL	Discri uni	48000	7	HC11, MGM, TG, NO, HE10, SDBD, SH	917	939	0.9613	0.9618	7	69	34	11	6	HE10
ALL	Discri multi	48000	6	HC11, MGM, TG, NO, HE10, SDBD	1095	789	0.9608	0.9544	8	33	16	8	6	
ALL	Discri uni	48000	6	HC11, MGM, TG, NO, HE10, SDBD	981	904	0.9607	0.9591	9	59	29	12	5	HE10
ALL	Gini	48000	5	HC11, MGM, TG, NO, HE10	856	1073	0.9598	0.9643	10	27	13	6	5	
ALL	Gini	48000	4	HC11, MGM, TG, NO	927	1006	0.9597	0.9614	11	31	16	7	4	
ALL	Discri uni	48000	5	HC11, MGM, TG, NO, HE10	974	1020	0.9585	0.9594	12					
ALL	Discri uni	48000	4	HC11, MGM, TG, NO	976	1018	0.9585	0.9593	12					
ALL	Discri multi	48000	5	HC11, MGM, TG, NO, HE10	1006	1006	0.9581	0.9581	14					
ALL	Discri multi	48000	4	HC11, MGM, TG, NO	929	1127	0.9572	0.9613	15					
ALL	Gini	48000	3	HC11, MGM, TG	963	1141	0.9562	0.9599	16					
ALL	Discri multi	48000	3	HC11, MGM, TG	869	1242	0.9560	0.9638	17					
ALL	Discri uni	48000	3	HC11, MGM, TG	1050	1069	0.9559	0.9563	18					
ALL	Discri multi	48000	2	HC11, MGM	986	1160	0.9553	0.9589	19					
ALL	Discri uni	48000	2	HC11, MGM	1015	1150	0.9549	0.9577	20					
ALL	Gini	48000	2	HC11, MGM	988	1188	0.9547	0.9588	21					
ALL	Discri uni	48000	1	HC11	1023	1163	0.9545	0.9574	22					
ALL	Discri multi	48000	1	HC11	1023	1163	0.9545	0.9574	22					
ALL	Gini	48000	1	HC11	988	1205	0.9543	0.9588	24					
ALL	Gini	48000	1	MGM	1814	1387	0.9333	0.9244	25					
ALL	Discri uni	48000	1	MGM	1588	1635	0.9329	0.9338	26					
ALL	Discri multi	48000	1	MGM	1588	1635	0.9329	0.9338	26					
